# IDENTIFYING AND MAPPING RURAL VETERANS’ GERIATRIC NEEDS AND RESOURCES

**DOI:** 10.1093/geroni/igae098.0978

**Published:** 2024-12-31

**Authors:** Porooshat Dadgostar, Lynette Kelley, Eileen Dryden, Lauren Moo, Orna Intrator, William Hung

**Affiliations:** University of Rochester, Rochester, New York, United States; Eastern Colorado Health Care System Rocky Mountain Regional VA, Aurora, Colorado, United States; Veterans Health Administration, Bedford, Massachusetts, United States; Department of Veterans Affairs, Bedford, Massachusetts, United States; Canandaigua VAMC and University of Rochester, Canandaigua, New York, United States; James J Peters VA Medical Center, James J Peters VA Medical Center, New York, United States

## Abstract

Veterans in rural areas often lack access to specialized geriatric care and may struggle to reach distant VA facilities for treatment. To better understand the regional needs of aging rural Veterans, this project aims to identify regional needs and resources for geriatrics services to guide future expansion efforts. We describe efforts to identify and map Veterans’ utilization of geriatrics services in three states with large rural populations (Wisconsin, Montana, and Hawaii) to help the VA improve targeting of these resources. By examining utilization data in these three states, we try to pinpoint local areas where there are gaps in services and where Veterans may have unmet geriatric needs. We utilized data from the Geriatric Extended Care Services Data Analysis Center (GECDAC), VA’s comprehensive population-based dataset for geriatric services. To develop these maps, we collected information on over 130,000 Veterans residing in the three states and obtained their sociodemographic variables and risk measures, including frailty, number of falls, Care Assessment Need (CAN) Score (estimated probability of hospitalization or death within 90 days or 1 year), dementia, long-term institutionalization risk, and use of Beers medications (potentially harmful medications for older adults). We observed the number of Veterans using different geriatric services, including geriatric ambulatory care, geriatric inpatient care, residential care, home and community-based care services, personal care services, facility-based care, and Hospice care. These data will form the basis of the “business case” for local leaders and the VA Office of Rural Health to expand geriatric services where they are most needed.

